# Barcoding pest species in a biodiversity hot-spot: the South African polyphagous broad-nosed weevils (Coleoptera, Curculionidae, Entiminae)

**DOI:** 10.3897/BDJ.9.e66452

**Published:** 2021-06-30

**Authors:** Steffan Hansen, Pia Addison, Laure Benoit, Julien M Haran

**Affiliations:** 1 Stellenbosch University, Stellenbosch, South Africa Stellenbosch University Stellenbosch South Africa; 2 University of Stellenbosch, Stellenbosch, South Africa University of Stellenbosch Stellenbosch South Africa; 3 CBGP, CIRAD, Montpellier SupAgro, INRA, IRD, Montpellier University, Montpellier, France CBGP, CIRAD, Montpellier SupAgro, INRA, IRD, Montpellier University Montpellier France

**Keywords:** Entiminae, PBNW, agricultural pests, identification, COI barcode

## Abstract

Polyphagous broad nosed weevils (Curculionidae: Entiminae) constitute a large and taxonomically challenging subfamily that contains economically significant agricultural pests worldwide. South Africa is a hot-spot for biodiversity and several species of indigenous and endemic genera of Entiminae have shifted on to cultivated plants, with some being phytosanitary pests. The sporadic pest status of many species (where the species has an occasional economic impact on the agricultural industry, but is not encountered often enough that is is readily recognisable by researchers and agricultural extension workers) and the presence of pest complexes and cryptic species represent an identification challenge to non-specialists. Furthermore, no comprehensive identification tools exist to identify immature stages that may be found in crops/soil. In this paper, a curated barcoding database with 70 COI sequences from 41 species (39 Entiminae, 2 Cyclominae) is initiated, to assist with the complexity of identification of species in this group.

## Introduction

Curculionidae Latreille, 1802 is a hyperdiverse family of beetles (Coleoptera), containing approximately 17 subfamilies and more than 51000 described species (Oberprieler et al. 2007, Bouchard et al. 2011, Leschen and Beutel 2014). Curculionidae are predominantly phytophagous and, as a family, utilise every organ of practically every higher plant species. Within the Curculionidae, the Entiminae Schönherr, 1823 is the subfamily with the highest species richness, containing approximately 54 tribes, 1280 genera and more than 13369 described living species (Oberprieler et al. 2007, Bouchard et al. 2011, Marvaldi et al. 2014, Yunakov 2020). Entiminae are usually small to medium-sized weevils (4-30 mm), with short, broad rostrums that are usually not more than twice as long as wide (Marvaldi et al. 2014). The adults have deciduous mandibular cusps that are used to dig their way out of soil after pupation and are then actively lost while feeding, leaving a distinctive scar (Thompson 1992, Marvaldi 1997, Marvaldi and Lanteri 2005). These deciduous cusps or the related scars in adult entimines are a commonly-used synapomorphy that establishes the monophyly of the subfamily; although it should be noted that it is not an autapomorphic feature, as it is shared by other subfamilies, possibly having evolved several times in Curculionidae or being secondarily lost by some taxa classified within Entiminae (Marvaldi 1997, Thompson 1992). Eggs of Entiminae are deposited directly on plant material and debris or soil, generally without using the rostrum in preparing the oviposition site (as is usual in other subfamilies in Curculionidae; Howden 1995, Marvaldi 1997) and the apodous, C-shaped larvae feed externally on plant roots, eventually pupating in soil (Marvaldi 1997, Marvaldi et al. 2014). Entimine larvae have the following synapomorphies: maxillary malae with four ventral setae; and a cushion-like, wider than long antennal sensorium (Marvaldi 1997) - this sensorium being elliptical from an apical view - mandibles with no accessory teeth on the intermediate portion of the cutting edge and mandibular scrobes that are lightly sclerotised and thus lighter in colour than the rest of the mandible (Marvaldi et al. 2018). The first two larval features are considered the most important synapomorphies for the monophyly of the Entiminae and are autapomorphies for the subfamily (Marvaldi 1998, Oberprieler et al. 2007, Marvaldi et al. 2018).

Most entimine weevil species have loose host plant associations, being oligo- or polyphagous in the larval and adult stages; several species constitute some of the most economically important agricultural pests worldwide (Oberprieler et al. 2007, Marvaldi et al. 2014). Larval feeding damages roots, whereas adult feeding damages leaves, shoots and fruits of their host plants, typically causing a notching pattern on the margins of leaves and shallow craters and/or scarring on fleshy tissue like fruits (Marvaldi et al. 2014, Prinsloo and Uys 2015). Parthenogenic reproduction and clonal lineages are not uncommon across Entiminae (and some Cyclominae Schönherr, 1826) tribes (Marvaldi et al. 2014). Some such species and lineages have become widespread pests globally, such as the South American *Pantomorus cervinus* Schönherr, 1840 and *Naupactus leacoloma* Boheman in Schönherr, 1840 (Entiminae: Naupactini; Marvaldi et al. 2014).

South Africa has a rich native fauna of Entiminae, notably with considerable species diversity across a number of tribes, including those such as Oosomini Lacordaire, 1863, Embrithini Marshall, 1942, Tanyrhynchini Schönherr, 1826 and Otiorhynchini Schönherr, 1826 that contain agricultural pest species (Schoeman 1983, Oberprieler 1988, Oberprieler 1995, Alonso-Zarazaga and Lyal 1999, Borovec and Oberprieler 2013, Hansen et al. 2020). The Entiminae includes agricultural pest species that have been recorded on every group of cultivated plants in the country (Marshall 1920, Marshall 1927, Marshall 1939, Annecke and Moran 1982, Oberprieler 1988, Prinsloo and Uys 2015). Many of the species recorded seem to be sporadic pests; despite having been recorded as pests in the previous century by Marshall (Marshall 1920, Marshall 1927, Marshall 1939), they do not feature as regularly occurring major pests of the crop on which they were originally recorded (Prinsloo and Uys 2015). Many native entimine pests (hereafter called ‘polyphagous broad nosed weevils’, PBNWs) probably shifted opportunistically from native vegetation on to a cultivated host as agriculture encroached on natural ecosystems, a process that has been deduced from pest appearance of *Eremnus cerealis* Marshall, 1921 (Entiminae: Tanyrhynchini) and *Oosomus varius* Boheman, 1843 (Entiminae: Oosomini) on small grains and vines and forestry pines (*Pinus radiata*, Pinaceae), respectively, in the Western Cape Province of southern Africa (Pettey 1922, Tribe 1991). Some species, like the banded fruit weevil, *Phlyctinus callosus* Schönherr, 1826 (Entiminae: Oosomini) are able to complete their life cycle in cultivated habitats (orchards and vineyards in South Africa), with larvae and adults feeding on crops and weeds and presenting a problem in the same area every growing season (Barnes 1987, Fisher and Learmonth 2003, Pryke and Samways 2007). In some cultivated habitats, like orchards and vineyards in the Western Cape Province of South Africa, several PBNW species may occur sympatrically (Magagula 2019). Some of these, like *Sciobius tottus* Sparrman, 1785 (Entiminae: Otiorhynchini), are consistently found in cultivated habitats, but only occasionally populations rise above the economic threshold, decreasing again in subsequent seasons (Pringle et al. 2015, Magagula 2019). A potential threat is posed by the polyphagous nature of PBNWs, as some may host shift on to crops that are novel and/or commercially expanding in South Africa to become major pests, such as on blueberries, Ericaceae (Bredenhand et al. 2010, Barnes et al. 2015).

Several PBNW species have successfully spread and established outside their native range, where they may become important pests. To current knowledge, three South African native species have become pests overseas. *Phlyctinus callosus* is a major introduced pest on vegetable and orchard/vineyard crops in New Zealand, Tasmania and Western Australia (Miller 1979, Butcher 1984, Sproul et al. 1986, Fisher and Learmonth 2003, Haran et al. 2020). *Sciobius tottus* has been recorded attacking *Pinus pinaster* (Pinaceae) on St Helena Island (Schoeman 1983) and *Afrophloeus squamifer* Boheman in Schönherr, 1843 (Entiminae: Embrithini) feeding on canola (Brassicaceae), medics (Fabaceae) and vetch (Fabaceae) in southern Australia (Borovec and Oberprieler 2013). A species of the genus *Systates* Gerstaecker, 1871 (Entiminae: Peritilini), a sub-Saharan African native genus (Alonso-Zarazaga and Lyal 1999), has recently been collected on an ornamental plant on Réunion Island, but has not been recorded as a pest in either its native nor introduced range and the genus’ host range is unknown (J. Haran, unpublished data). On the receiving end, the highly polyphagous South American Naupactini species *Naupactus leucoloma* (white-fringed weevil) and *Pantomorus cervinus* (Fuller’s Rose Weevil) and the *Medicago* (Fabaceae) feeding south-western European *Sitona discoideus* Gyllenhal, 1834 (Entiminae: Sitonini), have established successfully on their agricultural host plants in South Africa (Prinsloo and Uys 2015). Within South Africa itself, recent range expansions are observed between the south-western (Mediterranean) and north-eastern (subtropical) climatic regions (*Systates* sp., *Sciobius* spp. Schönherr, 1823; S. Hansen & J. Haran pers. obs.). As a result, South African crops may contain complex assemblages of species of PBNW for which species identification is challenging for non-specialists and an issue compounded by the presence of cryptic species in some taxa (Haran et al. 2020).

Barcode sequences of the mitochondrial gene cytochrome oxidase I (COI) have been shown to be an accurate and powerful tool in species identification of most animals, including insects (Hebert et al. 2003a, Hebert et al. 2003b). Identification with barcoding is based on reference sequences - the more extensive the reference the more accurate the tool becomes (Armstrong and Ball 2005, Floyd et al. 2010). Identification of pest insect taxa through barcoding has shown great potential as it is a fast, robust and accurate tool that requires relatively little tissue, may identify any life stage and has the potential to differentiate similar species that otherwise need a high degree of taxonomic expertise to identify (Armstrong and Ball 2005, Floyd et al. 2010, Germain et al. 2013, Sow et al. 2018). In some taxa, such as *Phlyctinus*, females do not have robust external features that allow for morphological species differentiation (Haran et al. 2020). Furthermore, the life history of many PBNW species still remains a mystery. A barcoding database will allow identification of eggs, larvae and pupae that might, as such, be linked to a specific plant and/or soil type. Barcode identifications allow any life stage or sex to be identified with the same level of certainty as adults (Floyd et al. 2010, Germain et al. 2013). The published COI barcodes of key PBNW species, together with properly photographed and curated voucher specimens of species placed in a museum, allow for a greater scope of study on the taxonomy, biology, distribution and host range of potential and recognised PBNW pests (Floyd et al. 2010, Germain et al. 2013).

This study initiates a curated barcode database of PBNW found in crops and disturbed habitats in South Africa to assist in rapid and robust identification of species, irrespective of sex or life stage of specimens.

## Material and methods

### Sampling

Specimens were collected alive from agro-ecosystems with recorded damage of weevils, disturbed roadside habitat and natural environments in South Africa (with focus on the Western Cape Province), between 2017 and 2020 (Suppl. material 1). Collecting permits were granted by landowners and by the appropriate agencies (see Acknowledgements). One specimen of the South African native genus *Systates* was obtained from Réunion Island and included in the study. An elevation map of the sampling sites was drawn up using QGIS 3.10 (Fig. 2). The collection method consisted of beating/sweep netting vegetation at night/early morning and visually searching at the base and debris on soil at the base of likely host plants during the day. All specimens were stored at ambient temperature in 96% ethanol until mounting and sequencing. Latitude and longitude were recorded, as well as host plant record, where possible. Only adult specimens were collected and used in this study.

Species were identified, based on external morphology using the keys and descriptions of Schoeman (1983), Oberprieler (1988), Borovec and Oberprieler (2013), Prinsloo and Uys (2015) and Haran et al. (2020). Reference collections housed at Iziko Museum (Cape Town, South Africa) and South African National Collection of Insects (SANC, Pretoria) were also consulted to cross-validate identifications with type material and specimens identified by specialists (Suppl. material 1). The male genitalia has high diagnostic value in Entiminae (Borovec and Skuhrovec 2018, Borovec 2019, Haran et al. 2020). For genera requiring the dissection of genital structures, the full abdomens were removed and soft tissue digested with potassium hydroxide (KOH) to obtain the genitalia (penis, copulatory sclerite and tegmen of males and sternite VIII, spermatheca and gonocoxites of females). All voucher specimens (including their genitalia stored in glycerol) were mounted on a card and deposited in the collections listed in Suppl. material 1. Two cyclomine weevil species, collected in disturbed agricultural habitats, are included in this study; a native unidentified species of *Rhyparosomus* Schönherr, 1842, (Cyclominae: Rhythirrinini) and the exotic vegetable weevil, *Listroderes costirostris* Schönherr, 1826 (Cyclominae: Rhythirrinini), which is a common pest on vegetables in the Western Cape Province of South Africa (Visser 2009). All of the weevil species in this study, apart from *Naupactus leucoloma*, *Pantomorus cervinus*, *Sitona discoideus* and *Listroderes costirostris*, are native to South Africa. When possible, multiple specimens per species were sequenced in order to estimate the level of intraspecific distances encountered in these taxa (Suppl. material 1).

#### DNA extraction, amplification and sequencing

The right hind leg of each prepared specimen was used for DNA extraction. The DNA was extracted using a DNeasy Blood & Tissue Kit (Qiagen, Hilden, Germany). PCR amplification was done for the COI standard barcoding region (Hebert et al. 2003b) of invertebrates using standard primers (traditional Folmer et al. 1994) that have been adapted by Germain et al. 2013 to a primer cocktail and M13-tails added, to increase amplification success and allow for sequencing, respectively (Ivanova et al. 2007;Table 1). PCR reactions were carried out in a Mastercycler Nexus (Eppendorf, Hamburg, Germany) with a final volume of 10 µl containing 5 µl of Multiplex PCR Master Mix (Qiagen, Hilden, Germany), 2 µM of each primer and 2 µl of DNA template. The PCR conditions were as follows: initial DNA denaturation at 94°C for 15 min, followed by 35 cycles of 30s at 94°C, 1 min at 52°C and 1 min at 72°C and a final extension of 15 min at 72°C. The PCR products were paired-end sequenced by Eurofins Genomics (http://www.eurofinsgenomics.eu/).

### Sequence analysis

The barcode sequences were aligned and manually checked using CodonCode Aligner ver. 3.7.1 (CodonCode Corporation, Centerville, MA, USA), verifying the absence of pseudogenes using standard detection methods (Haran et al. 2015). The sequences and GenBank codes obtained for multiple specimens of the six *Phlyctinus* species in Haran et al. 2020) are reported again in this study (Suppl. material 1, NCBI GenBank (https://www.ncbi.nlm.nih.gov/genbank/, codes MN627231-MN627250) and used as data in further sequence analysis. Pairwise sequence divergences were calculated using the Kimura-2-Parameter, K2P, (Kimura 1980) in MEGA7 (Kumar et al. 2016), utilising the ‘pairwise-deletion of gaps’ option (Suppl. material 2). Pairwise sequence divergence was visualised on a Neighbour-Joining (NJ) tree (Saitou and Nei 1987) using K2P distances in MEGA7. All specimens that showed a intraspecific K2P distances of ≥ 2% from their conspecifics are given a distinct haplotype (‘H’) differentiation (Suppl. material 1). This threshold was decided upon, based on the deep intraspecific variation shown by most species with multiple sequences obtained and thresholds used by other studies on Lepidoptera and Coleoptera (Bergsten et al. 2012, Mutanen et al. 2012). The flightless nature and, therefore, poor natural dispersal ability that may lead to high genetic subdivision between different populations of the same species (Peterson and Denno 1997) of most of the PBNW species in this study, also influenced the decision to use a 2% K2P threshold.

### Data resources

The collection and voucher data of all specimens used in this study, including identification of specimens, images, primer cocktails used in amplification, sequences and trace files are deposited at BOLD (Ratnasingham and Hebert 2007), the Barcode of Life Data System, under the project name CURSA,

http://dx.doi.org/10.5883/DS-CURSA1 (Hansen et al. 2020) and subsequently deposited in GenBank (https://www.ncbi.nlm.nih.gov/genbank/, codes MT674814-MT674861), Suppl. material 1 .

The COI sequence data of the *Phlyctinus* spp. (JHAR0941-0101, JHAR0755-0101, JHAR2288-0101, JHAR2290-0101, JHAR2086-0101, JHAR2101-0101, JHAR0822-0101, JHAR1353-0101, JHAR2252-0101, JHAR2252-0101, JHAR0819-0101, JHAR1016-0101, JHAR2173-0101, JHAR1304-0101, JHAR1195-0101, JHAR0973-0101, JHAR1300-0101, JHAR1082-0101, JHAR2264-0101) and *Oosomus* sp. (JHAR01073-0101) obtained in Haran et al. 2020) are deposited at NCBI GenBank (https://www.ncbi.nlm.nih.gov/genbank/), codes MN627231-MN627250 (Suppl. material 1).

The COI sequence data obtained by Magagula 2019 in a thesis at Stellenbosch University and sequences, originally obtained in the current study, are deposited at NCBI GenBank (https://www.ncbi.nlm.nih.gov/genbank/), codes MT674814-MT674861 (Suppl. material 1).

## Results

A total of 70 COI barcode sequences > 500 bp, from 41 morphospecies (39 Entiminae, 2 Cyclominae) are presented in this study. Of these, all have been identified to genus level, 29 to species level and 12 of the morphospecies were either not possible to identify or can not be identified with certainty (Suppl. material 1). No evidence was found of pseudogene amplification and no sequences were shared between species. A total of 50 new sequences from 35 morphospecies, 23 of which have been identified with certainty to species level, have been deposited in GenBank (GenBank codes MT674814-MT674861, Suppl. material 1). This includes previously unpublished sequences of PBNWs from vineyards and apple orchards in the Western Cape Province of South Africa, obtained by Magagula (2019) in a thesis. Only three specimens did not amplify (Suppl. material 1). All the COI sequences, except MN627242 (*Phlyctinus xerophilus* Haran, 2020, 615 bp), MN627249 (*Phlyctinus grootbosensis* Haran, 2020, 563 bp) and MT674827 (*Eremnus laticeps* Boheman, 1843, 649 bp) are 658 bp in length and include the 648 bp barcoding region for animals (Hebert et al. 2003b).

Of the 14 species for which more than one sequence was obtained, eight had K2P intraspecific variation ≥ 1%. Of these, five had intraspecific K2P variation of 2% or higher and were divided into haplotypes (Suppl. materials 1, 2). These species and their maximum intraspecific K2P distance obtained in this study are: *Afrophloeus spathulatus* Boheman in Schönherr, 1843 (6.1%), *Eremnus atratus* Sparrman, 1785 (2.8%), *Eremnus horticola* Marshall, 1920 (2.0%), *Phlyctinus callosus* (3.0%) and *Phlyctinus xerophilus* (9.2%). The specimens of *Systates* sp. (MT674859, MT674860), *Sciobius pollinosus* Fahraeus, 1871 (MT674854) and *Sciobius cf. pollinosus* (MT674853) (Suppl. material 1) are considered separate species in subsequent analysis, due to the specimens not being morphologically identifiable with certainty, due to a lack of appropriate identification tools and the K2P distance between the specimens of 11.2% and 9.7%, respectively.

Amongst the present dataset of barcode sequences, the mean intraspecific distance is 2.1% (max 9.2%, min 0.0%), the mean of the maximum intraspecific distances is 2.2% and the mean distance to the nearest heterospecific is 13.9%. The smallest interspecific distance is 4.3%. There is some overlap between intra- and interspecific distances and a small barcoding gap (mean distance to nearest heterospecific being 6.3-fold higher than the mean of maximum intraspecific distances) with this conservative method of calculation. Out of the 14 species for which two or more sequences were obtained, six had maximum intraspecific distances of ≤ 10% that of the distance to the closest heterospecific and 10 had intraspecific distances ≤ 12% that of the distance to the closest heterospecific. Except for *Phlyctinus xerophilus*, the distance to the nearest heterospecific was always larger than the maximum intraspecific value.

**Figure 1.**
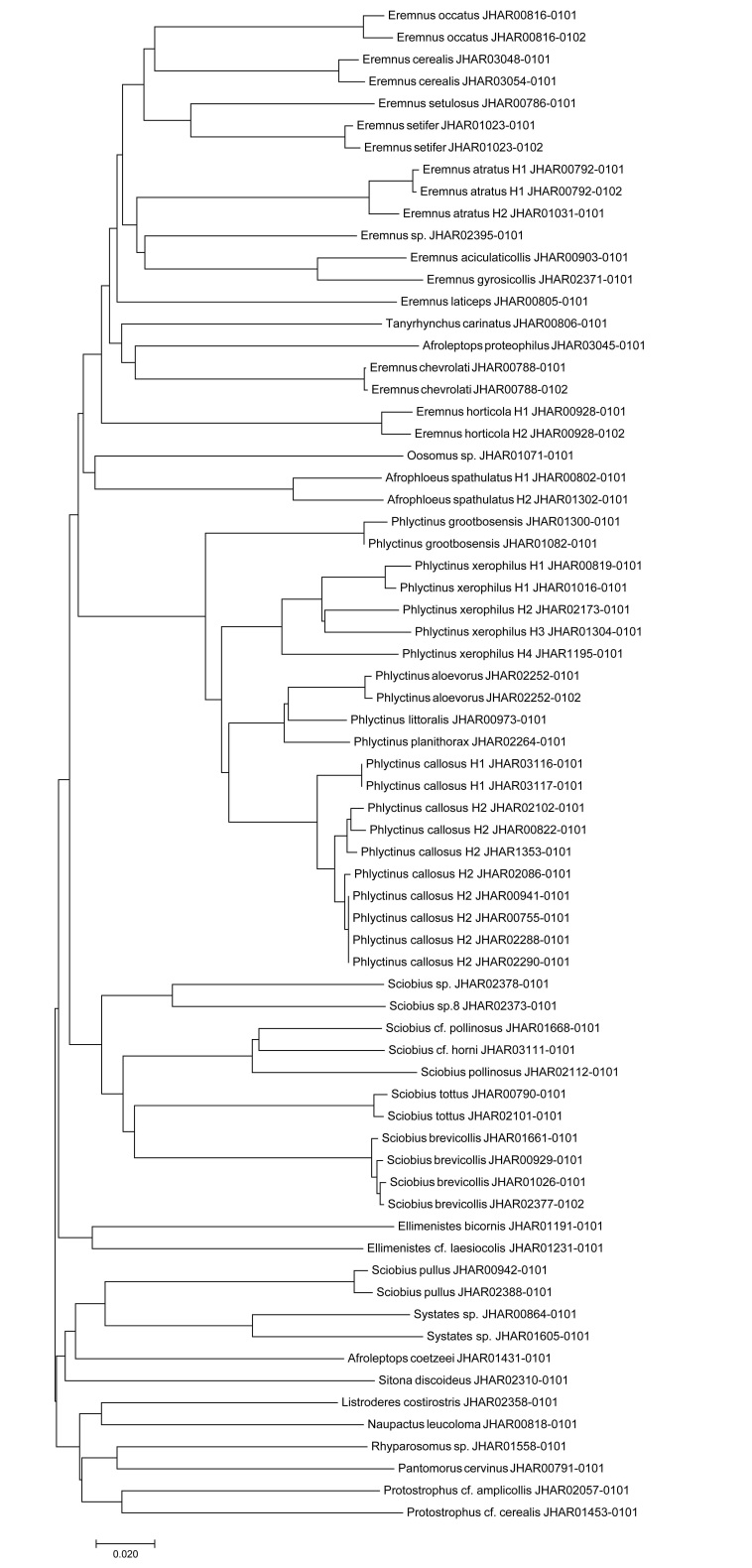
Neighbour-Joining tree of the COI sequences for polyphagous broad-nosed weevils (Coleoptera: Curculionidae) obtained/used in this study.

The NJ tree of the obtained sequences show distinct, non-monopheletic groupings amongst species in the genera *Eremnus* Schönherr, 1826, *Afroleptops* Oberprieler, 1988 and *Sciobius* and non-monopheletic groupings amongst genera and/or species in the tribes Tanyrhynchini (genera *Eremnus*, *Tanyrhynchus* Schönherr, 1826 and *Afroleptops* in this study), Embrithini (genera *Afrophloeus* Borovec and Oberprieler, 2013 and *Ellimenistes* Boheman, 1843, in this study) and Otiorhynchini (genus *Sciobius* in this study) (Fig. 1).

## Discussion

**Figure 2.**
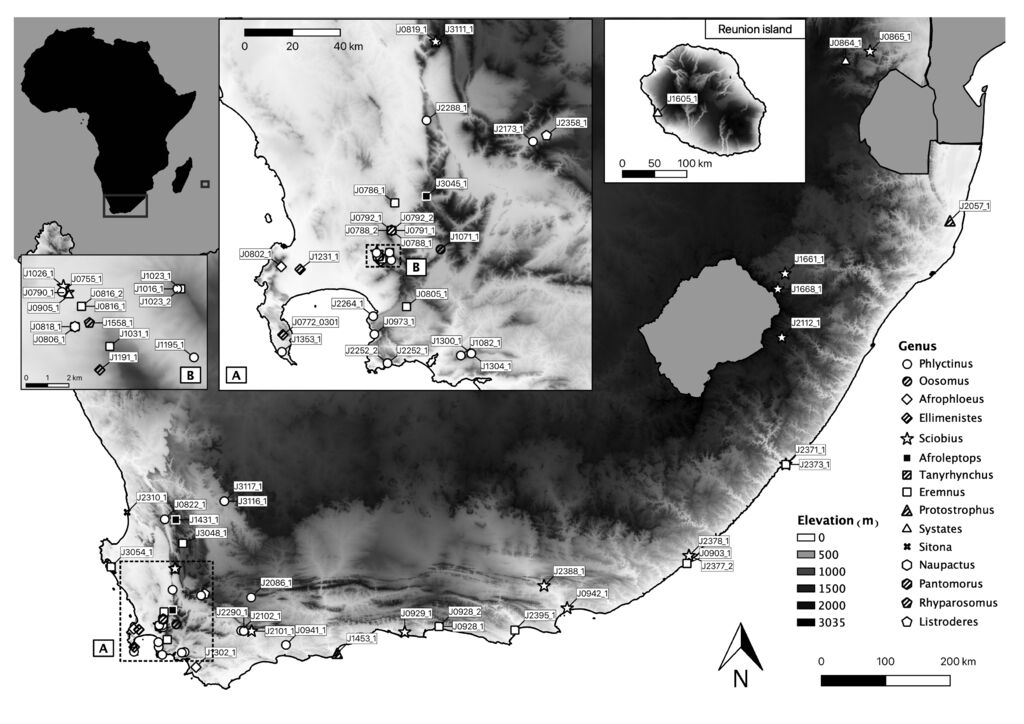
Map of sampling points of taxa used in this study collected from South Africa and Réunion Island, with (A) magnified insert of dense sampling points in south-western region of Western Cape Province and (B) Stellenbosch area.

Barcoding PBNW species from South Africa provides a valuable tool in rapidly and robustly identifying species of potential economic concern, including highly sporadic pest species that only rarely rise to population levels of economic concern. This study also reveals some challenges with the application of this approach to PBNW in South Africa. A small barcoding gap between the mean of maximum intraspecific genetic distance and the mean distance to closest heterospecific is observed, even without comprehensive sampling and multiple sequences per species for most of the PBNWs in this study. However, this result is not unexpected for Coleoptera (Meier et al. 2008, Bergsten et al. 2012) or, indeed, in other groups that have been well sampled (Meyer and Paulay 2005). The significant geographic distance between many of the sampled specimens in taxa, where multiple sequences were obtained, probably contributes to the high intraspecific genetic distances here observed (Fig. 2,Bergsten et al. 2012). This need however, not be an impediment to correct species identification using barcodes, provided adequate sampling and correct taxonomy of the group in question are undertaken (Ross et al. 2008). Deep intraspecific variation (often higher than 2%) and a ratio of largest intraspecific distance to nearest heterospecific distance often higher than 10% observed in this study will decrease the accuracy of identification (Ross et al. 2008). However, we believe the decrease in identification accuracy to be low enough to still allow for practical use, especially if the taxonomy of historically challenging groups like *Eremnus* and *Phlyctinus* (Oberprieler 1988, Haran et al. 2020) becomes better resolved.

The greatest challenge to successful barcoding of PBNW is undoubtedly inadequate taxonomic coverage of this group. The taxonomy of South African Entiminae is complex and many tribal/genus/species classification is still in the process of being resolved, with new species, genera and even tribes constantly being described (Meregalli et al. 2021). Recent examples from the past four years include 37 new species of *Pentatrachyphloeus* Voss, 1974 (Entiminae: Trachyphloeini); two species of *Heisonyx* Marshall, 1947 and two species of *Porpactus* Schönherr, 1842 (Entiminae: Embrithini); the new genera *Afromuelleria* Borovec and Skuhrovec, 2018 (Entiminae: Trachyphloeini) with four new species; and the new tribe Namaini Borovec and Meregalli, 2021 (Curculionidae: Entiminae) with six new genera and four new species being described (Borovec and Skuhrovec 2018, Borovec 2019, Borovec and Skuhrovec 2019, Meregalli et al. 2021). Some 'older' genera like *Eremnus* still contain a multitude of undescribed species (Oberprieler 1988). Some taxa consist of complexes of cryptic species that need a high degree of taxonomic expertise to identify (Haran et al. 2020) and are thus easily ‘overlumped’ (Funk and Omland 2003) by taxonomists. The dangers of inadequate taxonomy to a barcoding initiative are well illustrated by the genus *Phlyctinus*, which were long treated as a single species, *P. callosus*; recent taxonomic work has split this taxon into six closely-related species (Haran et al. 2020). Even now, the newly-named species *P. xerophilus* may itself constitute a complex of cryptic species (Haran et al. 2020, Suppl. material 1); if this proves to be the case, the barcoding gap observed in this study would increase and the overlap between intra- and interspecific distances would decrease. In the present study, about 36% of the species for which more than one sequence was obtained showed substantial genetic divergence (≥ 2% K2P variation) between intraspecific lineages. Although the sequencing of a complementary nuclear gene is required to determine if these lineages are reproductively isolated, such ratio gives a first indication on priority for future taxonomic treatments of pest species.

The NJ tree, calculated using the COI sequences, itself provides some preliminary signs that some South African genera and tribes, previously described only on morphological characteristics, might not form naturally monophyletic groupings (Fig. 1). It was already noted by Oberprieler (1995) and Borovec and Oberprieler (2013) that the tribes Embrithini and Tanyrhynchini and the genus *Eremnus* within Tanyrhynchini (Oberprieler 1988), may not form natural monophyletic groupings and are in need of comprehensive taxonomic revision and phylogenetic studies. It is surprising that all members of the genera *Sciobius* and *Afroleptops* sequenced did not group together in the NJ tree (Fig. 1), as these genera were considered monophyletic, based on morphological characteristics (Schoeman 1983, Oberprieler 1988). Although, conclusions should not be drawn from these results on a single gene region as further taxonomic and phylogenetic investigation into these taxa are warranted.

A further complication is produced by the potential presence of parthenogenic lineages in PBNWs in South Africa. Of the tribes treated here, Otiorhynchini and Naupactini are well known for containing species with parthenogenic lineages and the exotic species *Naupactus leaucoloma*, *Pantomorus cervinus* (Entiminae: Naupactini) and *Listroderes costirostris* (Cyclominae: Rhythirrinini) are confirmed parthenogenic species (Marvaldi et al. 2014). It has been demonstrated that parthenogenic lineages usually have a wider distribution range than diploid, sexual lineages and that sexual and parthenogenic lineages may co-occur in the same area (Normark 1996, Stenberg et al. 2004). Furthermore, deep COI divergences (4.0-7.3% in the Normark (1996) study of a Naupactini species complex), as well as morphological differences that are incongruent to COI differences between various reproductive lineages, have been observed for entimines (Normark and Lanteri 1998). Sampling was not extensive enough in this study to prove or disprove parthenogenesis in South African native taxa. The potential effects of COI and morphotype divergence that could be caused in species containing different reproductive lineages and the effect it may have on the success of a barcoding identification need to be considered as the PBNW barcoding tool is expanded. However, populations of all species sampled contained males, suggesting that sexual reproduction is prevalent amongst the PBNW sampled in this study.

The fourth challenge is insufficient sampling to adequately cover genetic diversity across the target groups' distributional range (Bergsten et al. 2012), especially within biodiversity hotspots for PBNWs, such as occur in the Western Cape Province (Oberprieler 1988, Haran et al. 2020). This may be compounded by the poor natural dispersal ability of typically flightless PBNWs, potentially allowing for relatively high genetic subdivision of different populations of the same species (Peterson and Denno 1997). This may potentially explain what is being observed in the *Systates* sp. from RSA and Réunion Island and *Phlyctinus callosus* from different areas within what is considered its native distribution, in this study.

A curated barcoding database (as on BOLD and GenBank) will enable the addition of sequences and species with every new study, allowing for taxonomic amendment. It can include location data, such that the robustness and accuracy in identifying specimens of the group of interest can continuously be improved (Armstrong and Ball 2005, Floyd et al. 2010). Although cost of barcoding identification of a sample may currently prove higher than a traditional morphological identification, barcoding identification is becoming increasingly standardised for use by researchers and agricultural extension services across the world. The cost of using barcoding for identification is predicted to decrease as technology advances and the current cost of barcoding identification is offset by the speed of identification (Floyd et al. 2010, Stein et al. 2014).

## Conclusions

The ability to accurately identify agricultural pest insects is key in their successful management. The PBNWs of South Africa are a diverse and taxonomically understudied group that contains a number of important pest species and strong potential for future pest emergence, due to their polyphagous nature. Their identification provides a challenge for non-specialists, an issue compounded by the presence of cryptic species in some taxa. The present curated barcode database provides a quick and simple identification tool that allows for a better understanding of their taxonomy, biology and distribution. This database aims at being continually expanded and improved as more species and specimens are sequenced and as taxonomic work progresses and improves current classifications, increasing the diagnostic power of barcode identifications in this challenging group.

## Acknowledgements

This project was funded in partial fulfilment of a thesis by HortGro Pome and Hortgro Stone and the South African Table Grape Industry (SATI). We thank the Iziko and SANC Pretoria Museum’s for the loan of valuable reference specimens, from which many of the identifications were made. We thank Noémie Hévin (CIRAD) for creating the sampling point map. This project would not have been possible without the hospitality of growers and land-owners who graciously allowed us to collect on their properties. Specimens were collected under permits from Cape Nature, Ezemvelo KZN Wildlife and Cape Research Centre SAN Parks; permits numbers CN44 30 4229, OP 1382/2019 and CRC/2019-2020/012--2012/V1, respectively.

## Supplementary Material

283A014B-F8B7-5145-96C6-1FF54D0C5CEF 10.3897/BDJ.9.e66452.suppl1 Supplementary material 1 Suppl. material 1 Data type BOLD and GenBank accession codes and collection data Brief description Voucher specimen BOLD codes, GenBank accession codes (where applicable) for COI sequences and collection data of the polyphagous broad-nosed weevils in the study File: oo_551754.xlsx https://binary.pensoft.net/file/551754 Steffan Hansen, Pia Addison, Laure Benoit and Julien Haran

65728299-674F-5A73-B3B5-A0B437031C2C 10.3897/BDJ.9.e66452.suppl2 Supplementary material 2 Supplementary 2. BDJ_15510 Data type K2P pairwise genetic distances Brief description The Kimura-2-Parameter pairwise genetic distances between COI sequences for polyphagous broad-nosed weevils obtained/used in study File: oo_506726.xls https://binary.pensoft.net/file/506726 Steffan Hansen, Pia Addison, Laure Benoit and Julien Haran

## Figures and Tables

**Table 1.** PCR primers and conditions. M13 tails from Ivanova et al. (2007) are in bold.

**Gene**	**Primer**	**Primer Sequence**	**Annealing temperature**	**Reference**
COI	HCO2198	**CAGGAAACAGCTATGAC**TAAACYTCDGGATGBCCAAARAATCA	52°C	Folmer et al. (1994), modified in Germain et al. (2013)
		**CAGGAAACAGCTATGAC**TAAACYTCAGGATGACCAAAAAAYCA		
		**CAGGAAACAGCTATGAC**TAAACTTCWGGRTGWCCAAARAATCA		
	LCO1490	**TGTAAAACGACGGCCAGT**TTTCAACTAAYCATAARGATATYGG		
		**TGTAAAACGACGGCCAGT**TTTCAACWAATCATAAAGATATTGG		
